# Minimum‐Phase Property of the Hemodynamic Response Function, and Implications for Granger Causality in fMRI

**DOI:** 10.1002/hbm.70285

**Published:** 2025-07-10

**Authors:** Leonardo Novelli, Lionel Barnett, Anil K. Seth, Adeel Razi

**Affiliations:** ^1^ School of Psychological Sciences and Monash Biomedical Imaging Monash University Melbourne Victoria Australia; ^2^ Sussex Centre for Consciousness Science, Department of Informatics University of Sussex Brighton UK; ^3^ CIFAR Program on Brain, Mind, and Consciousness Toronto Canada; ^4^ CIFAR Azrieli Global Scholars Program Toronto Canada; ^5^ Wellcome Centre for Human Neuroimaging University College London London UK

## Abstract

Granger causality (GC) is widely used in neuroimaging to estimate directed statistical dependence between brain regions using time series of brain activity. A known problem is that fMRI measures brain activity indirectly via the blood‐oxygen‐level‐dependent (BOLD) signal, which can distort GC estimates by introducing different time‐to‐peak responses across brain regions. However, how these distortions affect the validity of inferred connections is not fully understood. Previous studies have shown that false positives are not introduced if the haemodynamic response function (HRF) is minimum‐phase; but whether the HRF is actually minimum‐phase has remained contentious. Here, we address this issue by studying the transfer functions of three realistic biophysical models. We find that the minimum‐phase condition is met for a wide range of physiologically plausible parameter values. Therefore, statistical testing of GC can be viable even if the HRF varies across brain regions, with the following two limitations. First, the minimum‐phase condition is violated for parameter combinations that generate an initial dip in the HRF. Second, slow sampling of the BOLD signal (seconds) compared to the timescales of neural signal propagation (milliseconds) may still introduce spurious GC inferences. Beyond GC analysis, the closed‐form expressions for the transfer functions of these popular HRF models are valuable for modeling fMRI time series since they balance mathematical tractability with biological plausibility.

## Graphical abstract

The haemodynamic response variability across the brain can hinder Granger Causality analysis in fMRI. However, we show that three popular biophysical haemodynamic response models do not introduce false positives (their transfer functions are minimum‐phase) in most cases. The main limitation remains the slow fMRI sampling rate compared to neural timescales.

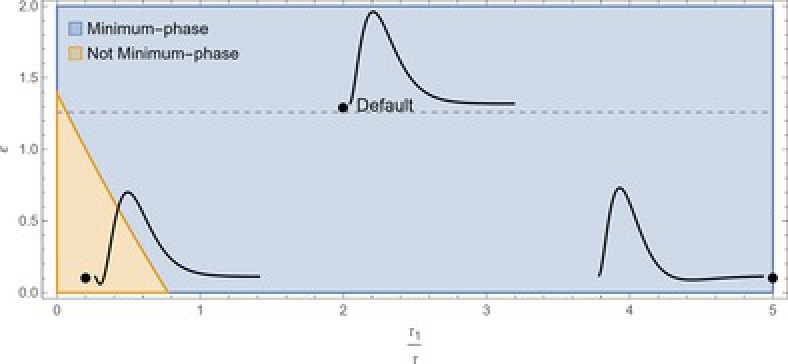

## 1 Introduction

The haemodynamic response function (HRF) is an important part of the biophysical models used in functional magnetic resonance imaging (fMRI). The HRF describes the temporal evolution of the blood oxygen‐level‐dependent (BOLD) signal in response to neural activity, mediated by vascular changes and neurovascular coupling. However, there is a lack of models that are both biologically realistic and mathematically tractable. Most HRF models in the literature are on opposite sides of the model complexity spectrum. The simple ‘canonical’ HRF model assumes that the response function is identical across the entire brain and employs a mixture of Gamma functions whose parameters lack physical interpretation (Glover 1999). Such simplicity allows one to model the BOLD signal as a convolution of neural activity with a fixed canonical HRF (Boynton et al. 1996), which guarantees mathematical tractability and simplifies model fitting (Frässle et al. 2021). On the opposite side of the spectrum, there are more realistic models based on physiologically‐informed nonlinear differential equations (Buxton et al. 1998; Friston et al. 2000; Stephan et al. 2007; Drysdale et al. 2010; Havlicek et al. 2015; Uludag and Havlicek 2021). Their parameters vary across brain regions and represent physiological mechanisms that regulate blood flow and oxygenation level.

Here, we linearise three of these popular biophysical models (Stephan et al. 2007; Drysdale et al. 2010; Havlicek et al. 2015) to make two contributions. First, the linearised HRFs fill the current gap in the model spectrum by balancing biological plausibility and mathematical tractability. They retain the biophysically interpretable parameters and their variation across the brain; yet, they still allow one to model the BOLD signal as a simple linear convolution and hence maintain analytical tractability. Second, we examine the properties of the linearised HRF models and their implications for the statistical testing of Granger causality (GC) estimates obtained from fMRI data.

GC was born in econometrics but has become an established analysis tool in neuroimaging to infer directed functional connectivity (Granger 1969; Seth et al. 2015; Cekic et al. 2018). In its simplest form, GC analysis considers two time series of brain activity (a source and a target) and asks whether observing the source improves the prediction of the target's activity compared to a prediction based on the target's past alone. The GC magnitude is an estimate of the reduction in prediction error. Exchanging the source and the target generally produces a different estimate, making GC a *directed* measure of statistical dependence, unlike correlation or mutual information. For Gaussian stochastic processes, GC is equivalent to the information‐theoretic transfer entropy, licensing an interpretation in terms of information flow (Barnett et al. 2009; Bossomaier et al. 2016). Traditional estimation of GC has relied on fitting discrete‐time autoregressive models to the data (Geweke 1984) and has focused on the one‐step‐ahead prediction error. Here, however, we will consider a more recent formulation of GC for continuous‐time processes (Barnett and Seth 2017), which enables the estimation of GC at any finite prediction horizon (i.e., time is modelled as a continuous variable).

The application of GC to fMRI is complicated because the HRF filters the neuronal signals and alters their temporal characteristics, distorting GC estimates (David et al. 2008; Deshpande et al. 2010; Smith et al. 2011; Handwerker et al. 2012; Wen et al. 2013; Barnett and Seth 2017). Thus, measuring GC using BOLD signals may yield a different result than using direct electrical recordings of neuronal activity. This issue prevents us from comparing the GC estimates. However, some important applications of GC do not rely on the GC magnitude but on its statistical significance against a null hypothesis (Kamiński et al. 2001; Cliff et al. 2021; Gutknecht and Barnett 2021). An important example is network inference (or ‘structure learning’ in Bayesian networks): building a statistical model of a system based on the network of directed relationships among its elements (Roebroeck et al. 2005; Novelli et al. 2019; Siggiridou et al. 2019). The critical question for the viability of network inference in fMRI is whether the HRF and its variability across brain regions introduce spurious relationships, that is, positive GC values between BOLD signals that are statistically significant—even if the GC between the corresponding neuronal signals is zero. Early studies found that GC inferences were not affected by a range of HRF variations, so long as sampling frequencies were sufficiently high (Seth et al. 2013; Wen et al. 2013). It has since been mathematically demonstrated that false positives are not induced, in principle, if the HRF is *minimum‐phase* (Solo 2016; Barnett and Seth 2017). This property requires both the HRF and its inverse to be causal and stable (the notion of minimum‐phase filter and the necessary conditions will be defined in the Methods section). However, whether the HRF is minimum‐phase has remained contentious (Solo 2016). Here, we address this issue by studying the transfer functions of three realistic biophysical models that are commonly used in the literature. We find that the minimum‐phase condition is met for a wide range of physiologically plausible HRF parameter values. Therefore, statistical testing of GC is viable even if the HRF varies across brain regions, with some notable limitations highlighted in the Section 4—in particular the requirement for fast sampling and the absence of an initial ‘dip’ in the HRF.

## 2 Methods

The hemodynamic response function (HRF) describes the temporal evolution of the BOLD signal in response to neural activity. This response is mediated by physiological processes involving vascular changes and neurovascular coupling. The HRF is influenced by several key factors: vasodilatory signaling, which initiates blood flow changes; cerebral blood flow; cerebral blood volume, particularly in the venous compartment; and changes in the local concentration of deoxyhemoglobin (Buxton 2012). Deoxyhemoglobin acts as an endogenous contrast agent due to its paramagnetic properties, which alter local magnetic field homogeneity and affect the MR signal. Typically, the HRF shows a delayed rise, peaking around 4–6 s post‐stimulus, followed by a return to baseline and often an undershoot. These dynamics reflect the complex interplay of blood flow, venous volume, and oxygen metabolism changes.

Biophysical models of the HRF aim to capture these interactions through a set of coupled differential equations whose parameters represent physiological properties such as vessel stiffness, blood transit time through the vascular bed, and resting oxygen extraction fraction. In this study, we examine three such biophysical models, each offering a different level of complexity in representing these physiological processes (Stephan et al. 2007; Havlicek et al. 2015; Drysdale et al. 2010). Different models involve different parameters, which we will introduce as they appear in the equations in Section 3. Some parameters are fixed based on current knowledge, while others are free, that is, they must be inferred from the data because they vary substantially across subjects or brain regions. In both cases, parameters are time‐invariant, that is, they do not depend on time. On the other hand, all three biologically detailed models involve dynamic (time‐evolving) variables that represent the following key processes and quantities:

- vasodilatory signal (s)
- blood flow (f)
- venous volume (v)
- deoxyhaemoglobin content (q).

These are grouped into a vector column variable $x={\left(s,f,v,q\right)}^{⊺}$ that evolves over time according to the haemodynamic state equation

(1) $$\dot{x}=F\left(x,u\right)$$

where u is the neuronal input that initiates the haemodynamic response. The function F varies between models, but the neuronal input generally induces vessel dilation and increases the incoming flow of oxygenated blood, leading to deoxyhaemoglobin content decay. The resulting BOLD change (y) is described by a second equation:

(2) $$y=G\left(x\right)$$

Here, F and G are nonlinear functions taken from three important haemodynamic models. For each model, our goal is to find the parameter ranges such that the HRF does not introduce false positives in the statistical significance testing of GC. As shown in previous studies (Solo 2016; Barnett and Seth 2017), this is guaranteed when the HRF meets the minimum‐phase conditions, which we explain in the next Section 2.1.

### 2.1 Minimum‐Phase Property of a Transfer Function

A function (or a ‘filter’ in signal processing) is *minimum‐phase* if and only if it is

- causal, that is, the output only depends on past and present values of the input,
- stable, that is, the response to a finite input is always finite,
- invertible, and such that its inverse is also causal and stable.

The mathematical definitions can be found in linear control theory textbooks, for example, Oppenheim et al. (1997). If all these conditions are met, the inverse system is also minimum‐phase. In other words, an inverse filter exists that is physically realizable, bounded, and can perfectly reverse the original system's effects. In practice, we will linearize the HRF equations and use the following equivalent definition of a minimum‐phase filter, which is easier to verify: a linear system is minimum‐phase if and only if all the zeros and poles of its transfer function have a negative real part. The concepts of a transfer function and its zeros and poles are briefly introduced in Section 2.2.

### 2.2 The Transfer Function

The transfer function is a core concept in signal processing and control theory for the frequency‐domain analysis of linear systems such as single‐input, single‐output filters. It describes the output amplitude for each input frequency and can be used to determine if a linear system is stable (does not diverge). To appreciate its utility in fMRI modeling, we can interpret the HRF as a filter that takes neuronal activity as the input and produces the BOLD signal as the output. The transfer function of the haemodynamic response describes the frequency spectrum of the BOLD signal as a function of the frequency spectrum of the neuronal input, where the spectrum is obtained via the Fourier transform (or, more generally, via the Laplace transform). We refer the reader to control theory textbooks for a thorough introduction and examples of transfer functions, for example, Oppenheim et al. (1997).

As anticipated in Section 2.1, testing the minimum‐phase condition is much simpler via the transfer function. The first step to obtaining the transfer function is to linearize the system of differential equations around their fixed point. Therefore, we will linearize each HRF model to obtain its state‐space representation^1^:

(3) $$\begin{array}{l} \dot{x}=Ax+\mathrm{Bu}\\ y=Cx \end{array}$$

The matrices A,B,C will then be used to compute the transfer function

(4) $$H\left(s\right)=C{\left(\mathrm{sI}-A\right)}^{-1}B$$

where s is the Laplace variable (Oppenheim et al. 1997). The transfer function fully characterises the linearised system and depends on the model parameters, which have a direct biological interpretation. These parameters are not indicated in the generic formula in Equation (4) but will appear explicitly once this is applied to specific HRF models in Section 3. In practice, $H\left(s\right)$ will be the ratio of two polynomials in the s variable (the numerator and the denominator), whose coefficients depend on the model parameters. The polynomial roots of the numerator (i.e., the values of s that make the denominator equal to zero) are called the *zeros* of the transfer function, while the roots of the denominator are called *poles*. We will find the zeros and poles of each of the transfer function of three HRF models to determine the parameter ranges that make each of them minimum‐phase according to the criterion provided in Section 2.1, that is, the parameter values such that all the zeros and poles of the transfer function have a negative real part. We will also use the transfer function to study the BOLD impulse response, that is, the response to an ideal, instantaneous neuronal input^2^, which is obtained via the inverse Laplace transform of the transfer function.

## 3 Results

### 3.1 Stephan et al. (2007) Model

The model proposed by Stephan et al. (2007) builds on the Balloon‐Windkessel equations and previous models (Buxton et al. 1998; Friston et al. 2000; Obata et al. 2004). Currently, this is the default HRF used in the SPM12 software (John Ashburner et al. 2020) for Dynamic Causal Modeling (Friston et al. 2003). It involves four hemodynamic state equations and one BOLD observation equation:

(5ad) Fx=−ks−γf−1+usf−v1/α/τf1−1−E01/fE0−qv1αv/τ

(5e) $$G\left(x\right)=\left(V_{0}\left(k_{1}\left(1-q\right)+k_{2}\left(1-q/v\right)+k_{3}\left(1-v\right)\right)\right)$$

#### 3.1.1 Interpretation of Equations and Parameters

This model consists of three parts, visually summarised in (Stephan et al. 2007, Figure 1). The first part describes the link between neural activity and regional cerebral blood flow using linear differential equations modelling a dampened oscillator [neurovascular state Equations (5a) and (5b)]. Specifically, the neural activity elicits an exponentially decaying vasodilatory signal s that drives blood flow f. In turn, the flow regulates s via a feedback mechanism (Friston et al. 2000). The parameter k in Equation (5a) is the signal decay rate, and γ is the feedback regulation rate (Table 1). Together, these parameters regulate the system to produce damped oscillations with a frequency of approximately 0.09 Hz. The flow f is normalised with respect to the resting flow, so the feedback regulation term $\left(f-1\right)$ in Equation (5a) becomes zero during rest.

**FIGURE 1:**
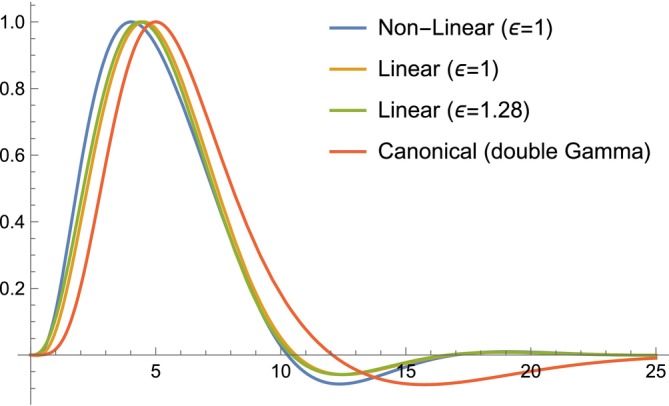
Impulse response of the biophysical model by Stephan et al. (2007) and its linearised version for different parameter values. The double‐Gamma ‘canonical’ response function is plotted for reference and all responses are rescaled to reach a peak value of 1.

**TABLE 1:** Parameter interpretation and default values in Stephan et al. (2007) and Havlicek et al. (2015).

Parameters and variables	Symbol	Value (Stephan et al. 2007)	Value (Havlicek et al. 2015)
Variables			
Vasodilatory signal	s		
Blood flow	f		
Venous blood volume	v		
Venous deoxyhemoglobin level	q		
Hemodynamic state variable	x	$x={\left(s,f,v,q\right)}^{⊺}$	$x={\left(s,f,v,q\right)}^{⊺}$
BOLD signal change (%)	y		
Input	u		
Neurovascular coupling parameters			
Decay of vasodilatory signal	k	(0.64)	0.6
Feedback regulation	γ	0.32	—
Gain of vasodilatory signal	ϕ	—	1.5
Decay of blood inflow signal	χ	—	0.6
Hemodynamic model parameters			
Mean transit time	τ	(2)	2
Vessel viscoelastic time	$\tau_{1}$	—	4
Balloon stiffness (Grubb's exponent)	α	0.32	0.32
Oxygen extraction factor at rest	$E_{0}$	0.4	0.4
BOLD model parameters			
Field strength (Tesla)	$B_{0}$	1.5	1.5
Echo time (s)	TE	0.04	0.04
Venous blood volume fraction at rest	$V_{0}$	0.04	0.04
Frequency offset at vessel surface for deoxygenated blood	$\theta_{0}$	40.3	40.3
Ratio of intra to extravascular signal	ε	(1.28)	0.1263–1.321
Sensitivity of intravascular signal relaxation rate to oxygen saturation	$r_{0}$	25	15
1st BOLD parameter	$k_{1}$	$4.3\theta_{0}E_{0}\mathrm{TE}=2.77$	$4.3\theta_{0}E_{0}\mathrm{TE}=2.77$
2nd BOLD parameter	$k_{2}$	$\varepsilon r_{0}E_{0}\mathrm{TE}=0.4\varepsilon$	$\varepsilon r_{0}E_{0}\mathrm{TE}=0.24\varepsilon$
3rd BOLD parameter	$k_{3}$	1−ε	1−ε

*Note:* The values in brackets are the Bayesian priors for the free parameters used in SPM12. The BOLD model parameter values are for a gradient‐echo sequence at 1.5 Tesla.

The second part is the hemodynamic model in Equations (5c) and (5d), which builds on the Balloon‐Windkessel equations and previous models (Buxton et al. 1998; Friston et al. 2000; Obata et al. 2004). It describes the behaviour of the post‐capillary venous compartment, modelled as an expandable balloon fed by the output of the capillary bed. The model consists of coupled equations representing mass balance for blood volume (v) and deoxyhemoglobin content (q) as they pass through the venous balloon, both normalised to their resting values. The blood volume change rate (v) is modelled as the difference between blood inflow and outflow in Equation (5c). The blood outflow function is based on Grubb et al. (1974)'s finding that the steady‐state relationship between cerebral blood flow and volume follows the power law $v^{1/\alpha }$, where α is the balloon stiffness (see Table 1). Similarly, the rate of change of deoxyhemoglobin (q) is modelled in Equation (5d) as the difference between the rate of delivery of deoxyhemoglobin into the venous balloon and its clearance rate from the tissue. The oxygen extraction fraction is modelled as a fixed nonlinear function of blood inflow, where $E_{0}$ is the net oxygen extraction at rest (Buxton et al. 1998). This reflects the physiological observation that a large increase in blood flow is required to support a small increase in oxygen metabolism. Both equations are scaled by the mean transit time of blood through the veins.

The third part of the model is the output or BOLD signal change in Equation (5e), which links venous volume (v) and deoxyhemoglobin content (q) to BOLD signal change (Buxton et al. 1998). This equation consists of three terms that respectively describe: (1) the extravascular signal as a function of deoxyhemoglobin content, (2) the intravascular signal, given by the ratio of deoxyhemoglobin to blood volume, and (3) the volume‐weighted balance between extravascular and intravascular signals. $V_{0}$ represents the resting blood volume fraction. The parameters $k_{1}$, $k_{2}$, and $k_{3}$ are dimensionless constants that reflect baseline physiological properties of brain tissue and acquisition parameters of the gradient echo sequence. These parameters depend on the magnetic field strength and other quantities detailed in Table 1, which also indicates their typical values. Key parameters in this equation include:

- ε: The ratio of intra‐ to extra‐vascular fMRI signal contributions. This value affects the overall BOLD signal amplitude and varies with field strength and vessel size.
- $\theta_{0}$: The field‐dependent frequency offset at the surface of a blood vessel for fully deoxygenated blood.
- $r_{0}$: The sensitivity (regression slope) of changes in intra‐vascular signal relaxation rate with changes in oxygen saturation (Buxton 2013; Obata et al. 2004).
- TE: The echo time, which influences the sensitivity of the BOLD signal to deoxyhemoglobin changes.

#### 3.1.2 Minimum‐Phase Analysis

The fixed point of the system in Equation (5) is $x_{0}={\left(0,1,1,1\right)}^{⊺}$ but one can perform the change of variables $x\to x-x_{0}$ so that the new fixed point is at the origin. Linearising the system around the origin yields the state‐space representation introduced in Equation (3), involving the following A,B,C matrices:

(6) $$\begin{array}{l} \dot{x}=\overset{A}{\overbrace{\left(\begin{array}{cccc} -k & -\gamma & 0 & 0\\ 1 & 0 & 0 & 0\\ 0 & \frac{1}{\tau } & -\frac{1}{\alpha \tau } & 0\\ 0 & \frac{E_{0}-\left(E_{0}-1\right)\log\left(1-E_{0}\right)}{E_{0}\tau } & \frac{\alpha -1}{\alpha \tau } & -\frac{1}{\tau } \end{array}\right)}}x+\overset{B}{\overbrace{\left(\begin{array}{c} 1\\ 0\\ 0\\ 0 \end{array}\right)}}u\\ y=\underset{C}{\underbrace{\left(0\;0\;V_{0}\left(k_{2}-k_{3}\right)\;V_{0}\left(-k_{1}-k_{2}\right)\right)}}x \end{array}$$

The transfer function of this state‐space model is obtained by plugging these matrices in Equation (4):
(7)

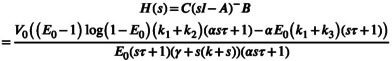

where s is the Laplace variable, and the other symbols are model parameters described in Table 1. The transfer function has four poles with negative real parts: $\left(-\frac{1}{\alpha \tau },-\frac{1}{\tau },\frac{1}{2}\left(-\sqrt{k^{2}-4\gamma }-k\right),\frac{1}{2}\left(\sqrt{k^{2}-4\gamma }-k\right)\right)$. The only zero of the function is $\frac{-2.50598\varepsilon -3.66903}{1.67231\tau \varepsilon -2.10961\tau }$, which has negative real part when ε>1.27. Therefore, this is the minimum‐phase condition. Finally, the haemodynamic response to an impulse (Dirac delta function $u\left(t\right)=\delta \left(t\right)$) can be obtained via the inverse Laplace transform of the transfer function. For the default parameter values used in SPM12 (the prior mean values used in Dynamic Causal Modelling), the haemodynamic response is plotted in Figure 1.

### 3.2 Havlicek et al. (2015) Model

Havlicek et al. (2015) proposed the following improved model:

(8) $$\begin{array}{l} F\left(x\right)=\left(\begin{array}{c} -\mathrm{ks}+u\\ \mathrm{\phi s}-\chi \left(f-1\right)\\ \left(f-\frac{\mathrm{f\tau}+\tau_{1}v^{1/\alpha }}{\tau +\tau_{1}}\right)/\tau_{1}\\ \left(\frac{f\left(1-{\left(1-E_{0}\right)}^{1/f}\right)}{E_{0}}-\frac{q\left(\mathrm{f\tau}+\tau_{1}v^{1/\alpha }\right)}{v\left(\tau +\tau_{1}\right)}\right)/\tau_{1} \end{array}\right)\\ G\left(x\right)=V_{0}\left(k_{1}\left(1-q\right)+k_{2}\left(1-q/v\right)+k_{3}\left(1-v\right)\right) \end{array}$$

All variables and parameters are explained in Table 1, and a visual summary is presented in Havlicek et al. (2017), Figure 1). One improvement with respect to (Stephan et al. 2007) concerns the balloon model: blood vessels are still modelled as balloons that inflate and deflate with increasing or decreasing blood flow; however, the balloon initially resists a change in blood volume, which better reflects empirical findings (Mandeville et al. 1998). Mathematically, this is achieved by adding a transient viscoelastic effect with a characteristic duration time $\tau_{1}$. Another improvement is the removal of the feedback mechanisms between the blood flow and the vasodilatory signal, which doesn't agree with experiments (Lindauer et al. 2010; Uludağ et al. 2004; Attwell et al. 2010).

**FIGURE 2:**
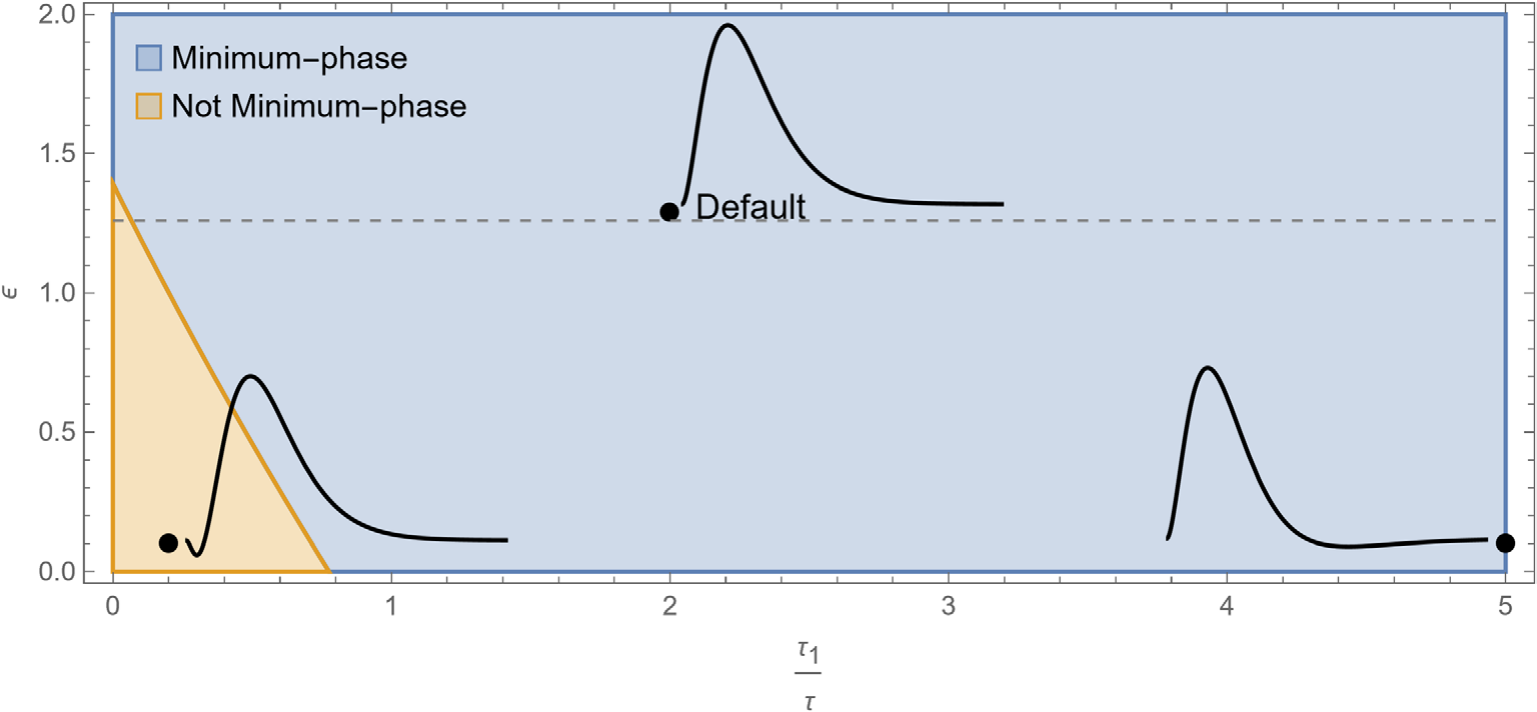
The hemodynamic response function obtained by linearising the biophysical model by Havlicek et al. (2015) meets the minimum‐phase conditions for a wide range of parameter combinations (blue region). The relevant parameters are $\frac{\tau_{1}}{\tau }$ (the ratio of vessel viscoelastic time to mean transit time) and ε (the ratio of intra‐ to extra‐vascular signal), as defined in Table 1. The three markers indicate the default parameters and two examples of parameter combinations that make the response minimum‐phase (right) or not minimum‐phase (left). For comparison, values above the dashed line also meet the minimum‐phase condition for the model by Stephan et al. (2007).

We use the same approach as in the previous section (a change of variable, followed by linearisation) to obtain the transfer function.

(9) $$\begin{array}{c} H\left(s\right)=\\ \frac{V_{0}\phi \left(\left(E_{0}-1\right)\log\left(1-E_{0}\right)\left(k_{1}+k_{2}\right)\left(\mathrm{\alpha s}\left(\tau +\tau_{1}\right)+1\right)-\alpha E_{0}\left(k_{1}+k_{3}\right)\left(s\tau_{1}+1\right)\right)}{E_{0}\left(s+k\right)\left(s+\chi \right)\left(s\tau_{1}+1\right)\left(\mathrm{\alpha s}\left(\tau +\tau_{1}\right)+1\right)} \end{array}$$

which has four poles with negative real parts: $\left(-\chi ,-k,-\frac{1}{\alpha \left(\tau +\tau_{1}\right)},-\frac{1}{\tau_{1}}\right),\frac{1}{2}\left(\sqrt{k^{2}-4\gamma }-k\right)$. The only zero of the function is $\frac{-3.75897\varepsilon -5.50355}{4.07905\tau +0.588471\tau \varepsilon +5.01694\varepsilon -6.32884}$, which has negative real part when τ or ε are sufficiently large. The minimum‐phase parameter region is visualised in Figure 2.

### 3.3 Drysdale et al. (2010) Model

This spatiotemporal BOLD model is derived from first principles using a poroelastic model of cortical tissue (Drysdale et al. 2010) and it uses the same BOLD change equation as the previous models Equation (5e). Its transfer function (Aquino et al. 2014) depends on several parameters that are explained in Table 2:

(10) $$\begin{array}{c} H\left(s\right)=\frac{C_{Z}\left(s+D/\rho_{f}\right)\left(k_{2}-k_{3}\right)}{s^{2}-2\Gamma s+\left(k^{2}+k_{z}^{2}\right)v_{\beta }^{2}}\times \frac{1}{-i{\left(s+\kappa /2\right)}^{2}+\omega_{f}^{2}}\\ \;\times \left\{1-\left(\frac{k_{1}+k_{2}}{k_{2}-k_{3}}\right)\frac{-V_{0}s+C_{Z}\left[\eta -\tau^{-1}\left(\beta -2\right)\right]}{s+\eta +\tau^{-1}}\right\} \end{array}$$

**TABLE 2:** Parameter interpretation and default values in Drysdale et al. (2010), as summarized in Pang et al. (2016)).

Parameter	Symbol	Value	Units
Blood flow signal decay rate	κ	0.65	$s^{-1}$
Flow‐dependent elimination constant	γ	0.41	$s^{-2}$
Blood mass density	$\rho_{f}$	1062	$\;\mathrm{kg}\;m^{-3}$
Effective blood viscosity	D	106−850	$\;\mathrm{kg}\;m^{-3}\;s^{-1}$
Mean elasticity exponent of cortical vessels	β	3.23	—
Haemodynamic transit time	τ	1	s
Fractional oxygen consumption rate	η	0.4	$s^{-1}$
Resting blood volume fraction	$V_{0}$	0.03	—
Field strength	$B_{0}$	3	T
Echo time	TE	0.03	s
Magnetic field parameters	$k_{1},k_{2},k_{3}$	4.2,1.7,0.41	—
Natural frequency of flow response	$\omega_{f}=\sqrt{\gamma -k^{2}/4}$	0.56	$s^{-1}$
Average cortical thickness	L	≈3	mm
Wave propagation speed	$v_{\beta }$	1−20	$\;\mathrm{mm}\;s^{-1}$
Wave damping rate	$\Gamma =1/2\left[D/\rho_{f}+C_{z}\left(\beta /\tau \right)\right]$	0.1−1	$s^{-1}$
Perpendicular spatial frequency	$k_{0}=\cos^{-1}\left(0.8\right)/L$	≈214	$m^{-1}$
Outflow normalization constant	$C_{z}=1\;\mathrm{mm}\times k_{0}/\sin\left({\mathrm{Lk}}_{0}\right)$	≈0.36	—
Spatial wavenumber in the cortical plane	$k^{2}=k_{x}^{2}+k_{y}^{2}$	>0	$\;m^{-2}$
Effective spatial frequency	$k_{z}=\sqrt{k_{0}^{2}+\left(1/v_{\beta }^{2}\right)C_{z}\left(\beta /\tau \right)D/\rho_{f}}$	≈214−950	$\;m^{-1}$

*Note:* The BOLD model parameter values are for a gradient‐echo sequence at 3 Tesla.

The five poles of the transfer function (Pang et al. 2016) are
(11)

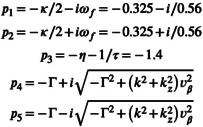




The first three have negative real part. For the remaining two poles, the square root is a real number because its argument is positive in the desired parameter range (see Table 2). Hence, the real part of the remaining two poles is

(12) $$-\Gamma =-\frac{D/\rho_{f}+C_{z}\beta /\tau }{2}=-0.5814-0.0005D$$

which is always negative since D is positive. The zeros of the transfer function are

(13) $$\begin{array}{l} z_{1}=-\frac{D}{\rho_{f}}=-\frac{D}{1062}\\ z_{2}=\frac{\left(k_{1}+k_{2}\right)C_{z}\left(\beta -\eta \tau -2\right)+\left(k_{2}-k_{3}\right)\left(\eta \tau +1\right)}{\tau \left(k_{2}\left(V_{0}-1\right)+k_{1}V_{0}+k_{3}\right)} \end{array}$$

The first zero has negative real part since D is positive (effective blood viscosity, see Table 2). The sign of the real part of the second zero depends on the BOLD change equation coefficients ($k_{1},k_{2},k_{3}$). With the choice of values in Table 2, we get $z_{2}=-3.2066$, which is negative, so the modelled HRF is minimum‐phase.

However, for consistency with the previous models, we can replace the fixed values of the BOLD equation coefficients ($k_{1},k_{2},k_{3}$) in Table 2 with the formulas in Table 1, which explicate their dependence on the physiological parameters ($\varepsilon ,\theta_{0},r_{0}$, TE) explained in Section 3.1.1. Now, the second zero of the transfer function ($z_{2}$) only has a negative real part when ε>0.9. Therefore, this constraint is the minimum‐phase condition for this HRF model.

### 3.4 Canonical HRF

The canonical HRF used in SPM12 is defined as a difference of two Gamma functions (John Ashburner et al. 2020; Lindquist et al. 2009):

(14) $$\frac{e^{-\frac{\mathrm{Tt}}{b_{1}}}{\left(\frac{\mathrm{Tt}}{b_{1}}\right)}^{a_{1}}}{\Gamma \left(a_{1}\right)\mathrm{Tt}}-\frac{e^{-\frac{\mathrm{Tt}}{b_{2}}}{\left(\frac{\mathrm{Tt}}{b_{2}}\right)}^{a_{2}}}{\Gamma \left(a_{2}\right)\mathrm{cTt}}$$

Unlike the previous biophysically realistic models, the parameters ($a_{1},a_{2},b_{1},b_{2},c,T$) that affect the shape of the HRF, for example, its peak value, time to peak, and duration (Handwerker et al. 2004) lack a direct biophysical interpretation in terms of blood flow, volume, or other physiological processes. The transfer function is obtained directly by taking the Laplace transform:

(15) $$H\left(s\right)=\frac{{\mathrm{cT}}^{a_{1}-1}{\left(b_{2}s+T\right)}^{a_{2}}-T^{a_{2}-1}{\left(b_{1}s+T\right)}^{a_{1}}}{c{\left(b_{1}s+T\right)}^{a_{1}}{\left(b_{2}s+T\right)}^{a_{2}}}$$

Substituting the default parameter values used in SPM12 ($a_{1}=6,a_{2}=16,b_{1}=16,b_{2}=16,c=6,T=16$), the canonical HRF assumes the familiar shape plotted in Figure 1, and its transfer function simplifies to

(16) $$H\left(s\right)=\frac{6{\left(s+1\right)}^{10}-1}{96{\left(s+1\right)}^{16}}$$

The only pole is at s=−1, and the 10 zeros have negative real parts, making the default canonical HRF minimum‐phase. Next, we vary the parameters in Equation (15) to test if the minimum‐phase conditions are still met. The zeroes and poles of the transfer function cannot be found analytically but were approximated numerically. Both have negative real parts for all tested parameters (default values ±3), making the canonical HRF minimum‐phase over a wide range of parameters.

## 4 Discussion

By linearising three popular biophysical HRF models (Stephan et al. 2007; Havlicek et al. 2015; Drysdale et al. 2010) and studying their transfer functions, we found the parameter ranges that make each linearised HRF minimum‐phase. This mathematical property is important because it ensures that the haemodynamic response doesn't introduce false positives in GC analysis, that is, positive GC values between BOLD signals that are statistically significant even if the GC between the neuronal signals is zero. In addition to these biophysical models, we studied the minimum‐phase properties of the ‘canonical’ HRF. This is an influential model that is widely used, for example, in the SPM software, although its parameters lack a direct biophysical interpretation. Our results show that

1. The canonical HRF is minimum‐phase for a wide range of parameter values, including the default ones used in SPM12.
2. The model by Stephan et al. (2007) involves several biophysical parameters, yet only one affects the minimum‐phase property (ε, the intra‐ to extra‐vascular signal ratio). The condition is ε>1.27, which is in the plausible physiological range reported in Table 1 (Havlicek et al. 2015; Uludağ et al. 2009).
3. The model by Havlicek et al. (2015) is minimum‐phase for plausible parameter ranges (ε, τ, and $\tau_{1}$) visualised in Figure 2.
4. The biophysical model by Drysdale et al. (2010) is minimum‐phase for ε>0.9.

In summary, all HRF models considered here are minimum‐phase for physiologically plausible parameter values. The parameter combinations that violate the minimum‐phase condition are those that generate an initial dip in the BOLD response (see Figure 2), confirming previous theoretical arguments (Solo 2016). Interestingly, the presence of an initial dip has long been debated but is yet unresolved. Early evidence based on optical imaging using 610–630 nm light has been disputed, and not many studies at 1.5 and 3 Tesla have reported an initial dip in the BOLD response (Uludağ 2010; Sirotin et al. 2009; Hillman 2014; Hong and Zafar 2018; Taylor et al. 2018), although it may be more pronounced at higher field strengths. The initial dip is also absent in the double‐Gamma canonical HRFs implemented in the FSL and SPM software, which are routinely used in fMRI data analysis Deshpande et al. (2010). Delayed HRF onset would also violate the minimum‐phase condition (Solo 2016). However, measurement noise makes it hard to distinguish an apparent delay from a slow ramping up of the HRF, and carefully designed studies have revealed that the hemodynamic response can detect neuronal events within milliseconds (Ogawa et al. 2000).

As noted above, the value of the intra‐ to extra‐vascular signal ratio (ε) needs further discussion. The choice of ε=1 in Stephan et al. (2007) was justified by the heuristic that this value is ‘roughly in the middle of the ε estimates at 1.5 T reported so far’. The authors noted that there is considerable uncertainty about the value of ε in the literature and this quantity is not well quantified because it is difficult to disambiguate intra‐ and extravascular BOLD signal experimentally. For example, Buxton et al. (1998) assumed ε=0.4 while Obata et al. (2004) estimated ε=1.43. This is why Stephan et al. (2007) didn't commit to one value for ε, unlike other parameters that were set to the fixed values as indicated in Table 1. Instead, they modelled this uncertainty in a Bayesian fashion by keeping ε as a free parameter with a prior distribution wide enough to encompass the range of physiological estimates. This allowed the posterior expectation to deviate from the prior mean while remaining positive. The prior mean was stated to be ε=1, but this is incorrect. For the estimate to always be positive, the authors used a log‐Normal distribution for ε. This is achieved by transforming a Normal random variable X (with mean μ=0 and variance $\sigma^{2}=0.5$) via the exponential function $\exp\left(X\right)$. The resulting distribution is plotted in Stephan et al. (2007, Figure 2), whose caption stated: ‘This figure shows a log‐normal probability density function (solid line) with a mean of 1 and a variance of 0.5’. However, perhaps unintuitively, the mean of this log‐Normal distribution is not simply $\varepsilon =\exp\left(\mu \right)=1$ as stated. The correct expression for the mean is $\varepsilon =\exp\left(\mu +\sigma^{2}/2\right)=1.28$. This value is above the threshold ε>1.27, which guarantees the HRF model by Stephan et al. (2007) is minimum‐phase according to our result in Section 3.1.2. Despite resolving this apparent discrepancy is important, we note that the difference between the HRF for ε=1 (below the minimum‐phase threshold) and ε=1.28 (above the threshold) is minimal, as can be seen in Figure 1. Thus, in practice, we wouldn't expect a significant increase in false positives. One could use simulations to precisely quantify any small discrepancies as a future extension of this work.

Another important question is whether it is possible to empirically determine if the HRF is minimum phase from the data. In some instances, the HRF for specific brain regions can be inferred by deconvolving the fMRI data, for example, if electrophysiological data is simultaneously acquired as a reference, or from task‐evoked BOLD responses. One could then assess if the estimated response presents features that violate the minimum‐phase condition, such as a pronounced initial dip or a delayed onset in the HRF. However, these features are subtle and challenging to establish from empirical observations due to measurement noise, as discussed above. An alternative approach is to estimate the HRF parameters by fitting a state‐space model to the data, as in spectral Dynamic Causal Modelling Razi et al. (2015); Novelli et al. (2024). While this relies on a model, the HRF parameters can be directly evaluated using the results presented herein, which provide the mathematical conditions under which three seminal HRF models are minimum phase.

Our study has two limitations. First, the minimum‐phase property is only tested for linearized HRF models, although a more general nonlinear theory of minimum‐phase systems exists (Byrnes and Isidori 1988). The fact that the minimum‐phase condition is not met for all parameter values, however, shows that it is not a trivial consequence of linearization per se. Second, slow sampling of the BOLD signal (in seconds) compared to the time scales of neural signal propagation (milliseconds) may still potentially introduce spurious GC (Solo 2016; Seth et al. 2013; Deshpande et al. 2010; Wen et al. 2013). Unfortunately, these limitations affect functional connectivity analysis in even greater measure: spurious correlations can arise even when the HRF is minimum phase, and even if the BOLD signal sampling rate were higher.

Beyond the implications for GC, the linearised HRFs are more broadly useful since they achieve a balance between complexity and tractability. They preserve the biophysically interpretable parameters and their variation across the brain; yet, they still allow one to model the BOLD signal as a simple linear convolution, maintaining analytical tractability. Fitting linearised HRF models to whole‐brain BOLD data can produce spatial maps of physiological processes, such as vasodilatory signal decay, transit time, and intra‐ to extra‐vascular signal ratio. Future studies can use them to reveal the vascular mechanisms behind existing maps of qualitative HRF features across the brain, for example, the peak magnitude or the time‐to‐peak (Chen et al. 2023; Taylor et al. 2018; Wu et al. 2013; Handwerker et al. 2004; Lindquist et al. 2009). These could also provide new insights into vascular changes that are prominent in aging and in neurodegenerative and psychiatric diseases (Tsvetanov et al. 2020; Iadecola 2004).

## Author Contributions

**Leonardo Novelli:** conceptualization, formal analysis, investigation, visualization, writing – original draft. **Lionel Barnett:** conceptualization; Writing – review and editing. **Anil K. Seth:** conceptualization; writing – review and editing. **Adeel Razi:** conceptualization; funding acquisition; supervision; writing – review and editing.

## Data Availability

Data sharing is not applicable to this article as no new data were created or analyzed in this study.

## Endnotes

^1^
Unlike typical state‐space models used in GC and DCM, here x denotes the haemodynamic state variable (not the neuronal state variable) and u denotes the neuronal activity (not the experimental input). The neuronal activity is the input that initiates the haemodynamic response.

^2^
Studying the impulse response to an ideal, instantaneous input (a Dirac delta function) is standard practice in control theory. In this context, we are modelling a notional neuronal input, not an external experimental input as in task‐based fMRI studies.
